# And now for something completely different: Inattentional blindness during a Monty Python's Flying Circus sketch

**DOI:** 10.1068/i0706sas

**Published:** 2015-01-22

**Authors:** Richard Wiseman, Caroline Watt

**Affiliations:** Psychology Department, University of Hertfordshire, Hatfield, Hertfordshire, UK; e-mail: r.wiseman@herts.ac.uk; Psychology Department, University of Edinburgh, Edinburgh, UK; e-mail: caroline.watt@ed.ac.uk

**Keywords:** inattentional blindness, perception, illusion, attention, Monty Python

## Abstract

Perceptual science has frequently benefited from studying illusions created outside of academia. Here, we describe a striking, but little-known, example of inattentional blindness from the British comedy series “Monty Python's Flying Circus.” Viewers fail to attend to several highly incongruous characters in the sketch, despite these characters being clearly visible onscreen. The sketch has the potential to be a valuable research and teaching resource, as well as providing a vivid illustration of how people often fail to see something completely different.

“Inattentional blindness” occurs when individuals fail to perceive something obvious and unexpected because their attention is engaged elsewhere. Researchers have long had an interest in inattentional blindness, with, for example, Münsterberg (1908) and Cornell (1959a, 1959b) reporting early demonstrations of the phenomenon (see Simons, 2011). In the 1970s, Neisser carried out systematic research into the topic, developing a paradigm in which observers attending to events in one of two superimposed films frequently failed to notice unexpected events in the other film (Neisser, 1979; Neisser & Becklen, 1975). More recently, researchers have built on Neisser's groundbreaking work and carried out a series of innovative studies into inattentional blindness (see, e.g., Hyman, Boss, Wise, McKenzie, & Caggiano, 2009; Simons and Chabris 1999). Interest in inattentional blindness also extends beyond academia, with magicians, artists, and filmmakers using the phenomenon to influence observers' perception (see, e.g., Kuhn, Amlani, & Rensink, 2008).

Despite widespread interest in inattentional blindness, most researchers are unaware that a striking example of the phenomenon appears in the British comedy series “Monty Python's Flying Circus.” Episode 12 of the second series of Monty Python's Flying Circus aired in December 1970 contained a short sketch entitled “Ypres 1914—Abandoned.” This sketch takes place during the First World War, and begins with a close-up image of a harmonica being played by a British soldier (Eric Idle). The camera then slowly zooms out to reveal four soldiers (including John Cleese, Eric Idle, and Michael Palin) sitting in an army encampment. Standing behind them are several actors dressed in a series of highly incongruous costumes, including that of a nun wearing a large white hat (Graham Chapman), a sheikh, and a Greek Orthodox priest. The film then cuts to a shot of the soldiers from another angle, and additional incongruous characters can be seen behind them, including a Viking and a topless man wearing a vibrant blue “Hawaiian” skirt. The incongruous characters are visible during the scene, with the nun being especially prominent (see Figure 1). Two of the soldiers (Idle and Palin) chat for about 20 s before an actor playing the role of a floor manager (Terry Jones) walks into shot, and asks anyone who is not involved in the scene to leave the set. The various incongruous characters then walk out of the scene and the audience laughs. The sketch lasts about 1 min 15 sec.

**Figure 1.**
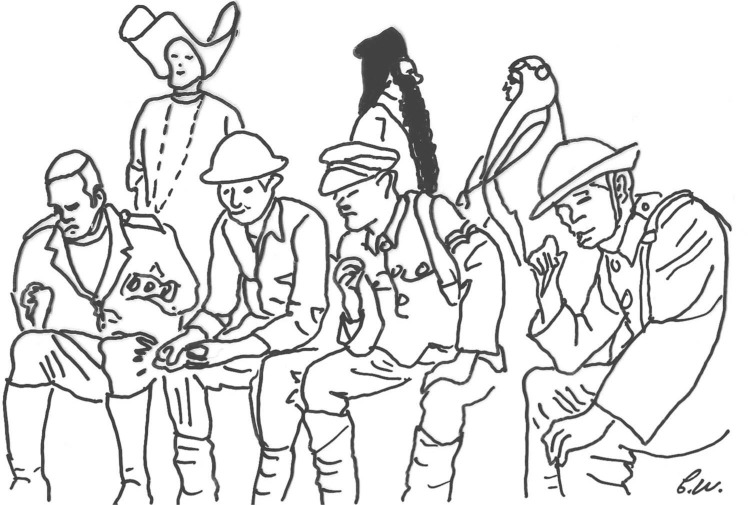
An outline drawing of a still frame from Monty Python's Flying Circus “Ypres 1914—Abandoned” sketch.

We showed the sketch to a group of undergraduates (*N* = 54) to discover the percentage of people that fail to perceive the incongruous characters, despite them being in full view throughout. The sketch was shown via a projector (image approximately 2 m wide) and participants watched en masse in a classroom setting. Immediately after viewing the film, participants were presented with a two-item questionnaire. First, participants were asked “Did you notice any of the unusual characters (e.g., the nun, priest, or Viking) before they walked out of the scene?” (Response options: “Yes,” “No”). 70.4% of participants failed to notice the incongruous characters prior to them leaving the scene. Second, those participants who had failed to notice the incongruous characters were asked “Are you surprised that you didn't notice any of the unusual characters before they walked out of the scene?” (Response options: “Yes”, “No”). 78.9% of participants expressed surprise that they hadn't spotted the characters. In short, as expected, the sketch produces a strong inattentional blindness effect.

The Python team may have intentionally created the sketch as a funny and clever perceptual illusion, a metaphor for how those involved in the First World War were often blind to the larger context of the conflict, or simply a piece of surreal humor. Either way, just as research into other illusions created outside of academia have proved beneficial to perceptual scientists (see, e.g., the “cafe wall illusion” [Gregory & Heard, 1979] and conjuring [Lamont & Wiseman, 1999]), the sketch has the potential to act as a useful teaching and research resource. For example, to help identify whether the initial focusing on the harmonica played an important role on the subsequent inattentional blindness, we created a new edit of the sketch that cut directly to the wide-shot containing the incongruous characters, and then showed the amended clip to another group of undergraduates (*N* = 74). The sketch was shown under the same conditions as before (e.g., approximately 2 m wide projected image, observed en masse in a classroom setting) and participants were presented with the same questionnaire immediately afterward. 62.1% of participants still failed to see the incongruous characters prior to them leaving the scene, and there was no significant difference in detection rates with and without the initial close-up shot (Chi-square = 0.93, *p*(2 − *t*) = 0.33), suggesting that the inattentional blindness effect produced by the sketch does not rely on the initial focusing. Additional work could examine other potential factors, including, for example, the sense of narrative and the soldiers chatting.

In short, it's hoped that Monty-Python's “Ypres 1914—Abandoned” sketch will now act as a useful teaching and research resource, and illustrate how we can all sometimes fail to see something completely different.
